# Efficacy and safety of tacrolimus versus corticosteroid as initial monotherapy in adult-onset minimal change disease: a meta-analysis

**DOI:** 10.1007/s11255-022-03122-7

**Published:** 2022-01-31

**Authors:** Jingkui Lu, Zhongxiu Xu, Wei Xu, Lifeng Gong, Min Xu, Weigang Tang, Wei Jiang, Fengyan Xie, Liping Ding, Xiaoli Qian

**Affiliations:** 1 Department of Nephrology, Wujin Hospital Affiliated With Jiangsu University, Changzhou City, 213000 Jiangsu Province China; 2 Department of Nephrology, The Wujin Clinical College of Xuzhou Medical University, Changzhou City, 213000 Jiangsu Province China

**Keywords:** Tacrolimus, Corticosteroid, Minimal change disease, Meta-analysis

## Abstract

**Objective:**

The objective of this meta-analysis was to compare the efficacy and safety of tacrolimus (TAC) monotherapy versus corticosteroid as initial monotherapy in adult-onset minimal change disease (MCD) patients.

**Methods:**

Databases including PubMed, Embase, the Cochrane Library, China National Knowledge Infrastructure, and Wanfang database were searched from the inception to March 20, 2021. Eligible studies comparing TAC monotherapy and corticosteroid as initial monotherapy for adult-onset MCD patients were included. Data were analyzed using Review Manager Version 5.3.

**Results:**

Four randomized controlled trials (RCTs) involving 196 patients were included in the meta-analysis. For initial monotherapy for adult-onset MCD, TAC and corticosteroid had similar complete remission (OR 1.06, 95% CI 0.47–2.41, *P* = 0.89), total remission (OR 1.30, 95% CI 0.39–4.35, *P* = 0.67), relapse rate (OR 0.63, 95% CI 0.28–1.42, *P* = 0.26). Main drug-related adverse effects of two therapeutic regimens had no difference concerning infection (OR 0.54, 95% CI 0.23–1.27, *P* = 0.15), glucose intolerance (OR 0.55, 95% CI 0.16–1.84, *P* = 0.33) and acute renal failure (OR 1.37, 95% CI 0.36–7.31, *P* = 0.71).

**Conclusion:**

TAC monotherapy is comparable with corticosteroid monotherapy in initial therapy of MCD. To further confirm the conclusion, more large multicenter RCTs are necessary.

## Introduction

Minimal change disease (MCD) causes up to 10–15% of primary nephrotic syndrome (NS) in adults [1]. Persistent NS results in infections, thromboembolic events, hyperlipidemia, cardiovascular disease [2–4]. Acute kidney injury accompanies the presentation of MCD in up to 20–25% of cases [5]. High doses of corticosteroid have been recommended as initial therapy of MCD according to Kidney Disease Improving Global Outcomes (KDIGO). Compared with children, adults often have a delayed response to corticosteroid [6]. The relapse of adult-onset MCD is over 50%. One-third of patients become frequent relapse or steroid-dependent [7]. This necessitates repeated or long-term use of corticosteroid. However, the adverse effects of high-dose corticosteroid also become common, which include in cosmetic changes, weight gain, impaired glucose tolerance, osteoporosis, infection, and gastrointestinal bleeding [8, 9].

Tacrolimus (TAC), an immunosuppressive macrolide of calcineurin inhibitors (CIN) group, is a relatively attractive alternative to corticosteroid for treatment of MCD. TAC can suppresses IL-2 transcription, inhibit the growth and differentiation of T cells, thereby reduce the immune damage of podocyte finally [10]. Compared with another calcineurin inhibitors such as ciclosporin, TAC showed stronger immunosuppressive effect and fewer side effects [11–13]. Some studies reported successful TAC treatment of frequently relapsing, steroid-dependent, and steroid-resistant MCD [14, 15]. However, compared with corticosteroid, the efficacy of TAC monotherapy as the first-line initial agent in adult-onset MCD is uncertain. Our meta-analysis was conducted to compare the efficacy and safety between TAC with corticosteroid in initial monotherapy of adult-onset MCD.

## Materials and methods

### Search strategy

We searched PubMed, Embase, the Cochrane Library, China National Knowledge Infrastructure, and Wanfang database from the inception to March 20, 2021. The combined text and MeSH terms included minimal change disease, corticosteroid, and tacrolimus. In addition, the cited papers and relevant references were searched manually to identify eligible studies. There was no language restrictions.

### Inclusion criteria

The inclusion criteria were defined as follows:

1. Randomized controlled trials (RCTs), cohort or case–control studies;
2. Age > 18 years, NS, a kidney biopsy showing MCD and serum creatinine level of < 133 μmol/L;
3. Studies were designed to compare TAC with corticosteroid as initial monotherapy for adult-onset MCD;
4. The main endpoint of the review was complete remission (CR) and total remission (TR). Secondary endpoints were relapse and drug-related adverse effects. CR is defined as proteinuria < 0.3 g/day with normal serum albumin and creatinine. Partial remission (PR) is defined as proteinuria 0.3–3.5 g/day which had declined to ≤ 50% of the baseline value. TR is defined as either CR or PR. Relapse is defined as proteinuria > 3.5 g/d in patients who had achieved CR or PR.

### Exclusion criteria

The exclusion criteria were defined as follows:

1. Case series, comments, reviews;
2. Lack of relevant outcomes data;
3. secondary minimal change disease, malignant tumour, infection (hepatitis B or C virus infection, tuberculosis and syphilis), diabetes mellitus, pregnancy, lactating, active gastrointestinal bleeding, other untreated infections, or any condition that would cause the study to be detrimental to the patient;

### Data extraction and quality assessment

Data were extracted independently by two investigators using standard data extraction forms. In the case of disagreement, a third investigator was consulted. We extracted characteristics including first author, year of publication, location, study design, follow-up period, age, sex, sample size, specific drug treatment program, and outcomes. The Cochrane assessment tool was used to evaluate the quality of RCTs [16].

### Statistical analysis

We performed the data analysis using Review Manager Version 5.3 (Cochrane Collaboration). Heterogeneity between studies was assessed using *I*^2^ statistics. We considered *I*^2^ > 50% and *P* < 0.10 to imply significant heterogeneity. Homogeneous data were performed using the fixed-effects model. Heterogeneous data were performed using the random-effects model. We presented categorical variables as Odds Ratios (OR). Summary estimates and 95% confidence intervals (CIs) were calculated. Overall effects were determined by the using *Z* test. A *p* value < 0.05 was considered significant. Publication bias was assessed using sensitivity analysis.

## Results

### Study selection and characteristics

**Fig. 1:**
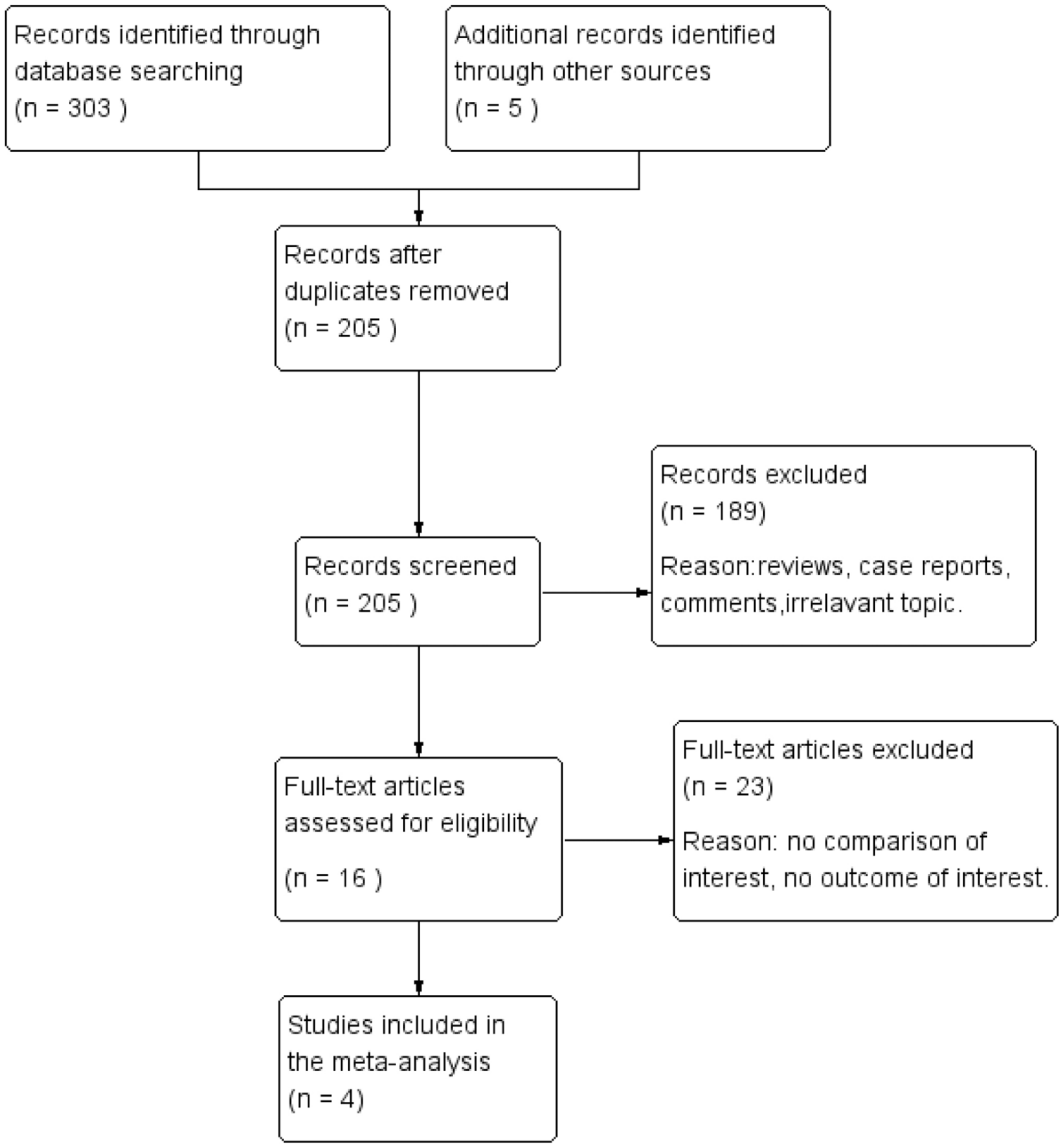
Flow diagram of the literature search

A flow diagram of the selection process is shown in Fig. 1. Finally, four RCTs were included in this analysis [17–20]. As a whole, 100 patients were included in TAC monotherapy group and 96 patients were included in steroid group. The follow-up period was from 36 to 82 weeks. The risks of bias in included RCTs were moderate. The baseline characteristics of these studies are listed in Table 1. Specific drug treatment program are listed in Table 2. The Cochrane assessment is listed in Table 3.

**Table 1:** Characteristics of the included studies

Study (year)	Country	Study design	Sample size	Follow-up period	Mean age (years)	Male/Female	SCr (μmol/L)	Proteinuria (g/day)	uPCR (mg/g)	Serum albumin (g/l)	Systolic BP (mmHg)	Diastolic BP (mmHg)	ACEI and/or ARB treatment
Li J (2012) [17]	China	RCT	TAC group:21	48 weeks	42.1 ± 20.2	3/4	85.1 ± 17.9	6.1 ± 3.7	-	20.4 ± 4.9	118.4 ± 12.4	73.6 ± 6.7	+
			Steroid group:20		46.6 ± 20.7	11/8	84.4 ± 11.5	7.1 ± 3.2		21.5 ± 5.6	121.8 ± 13.2	76.4 ± 6.4	
Li XY (2012) [18]	China	RCT	TAC group:27	36 weeks	–	–	–	–	–	–	–	–	?
			Steroid group:28										
Patil (2019) [19]	India	RCT	TAC group:25	18 months	28 ± 8	15/10	79.6 ± 17.6	5.1 ± 1.9	-	25 ± 0.5	109 ± 19	66 ± 8	+
			Steroid group:23		28 ± 7	15/8	88.4 ± 26.5	5.3 ± 2.0		22 ± 0.4	104 ± 15	73 ± 10	
Thomas (2020) [20]	United Kingdom	RCT	TAC group:27	82 weeks	43	12/15	72.5	–	7717	15	126	73	+
			Steroid group:25		39	15/10	71.6		6504	17	128	80	

*SCr* Serum creatinine; *uPCR* Urine protein-to-creatinine ratio; *BP* blood pressure; *ACEI* Angiotensin-converting enzyme inhibitor; *ARB* Angiotensin II subtype 1 receptor blocker; * +*  Patient was treated by ACEI and/or ARB; ? No description

**Table 2:** Specific drug treatment regimens

Study	Treatment time of TAC	TAC therapy regimens	Treatment time of steroid	Steroid therapy regimens
Li J (2012) [17]	48 weeks	Oral TAC was administered at a dose of 0.05 mg/kg/d (T0 levels of 5–8 ng/mL in the first 24 weeks and T0 levels of 3–6 ng/mL in the second 24 weeks)	48 weeks	Oral steroid was administered at a dose of 1 mg/kg/d (a maximum dose of 60 mg/d)for 8 weeks and tapered 5 mg every week to 30 mg/d, which was maintained for 2 months, and tapered gradually to 10 mg/d maintained for the end
Li XY (2012) [18]	36 weeks	Intravenous use of methylprednisone was given in the first 10 days. Oral TAC was administered at a dose of 1–2 mg/d (T0 levels of 5–10 ng/mL) and maintained for 2 months after CR, and tapered gradually (T0 levels of 3–8 ng/mL)	36 weeks	Intravenous use of methylprednisone was given in the first 10 days. Oral steroid was administered at a dose of 1 mg/kg/d and maintained for 2 weeks after CR, and tapered gradually
Patil (2019) [19]	12 months	Oral TAC was administered at a dose of 0.075 mg/kg/d (T0 levels of 8–10 ng/mL) and maintained for 3 months after CR, and tapered to 0.05 mg/kg/d (T0 levels of 4–8 ng/mL)until total duration of 12 months	6 months	Oral steroid was administered at a dose of 1 mg/kg/d (a maximum dose of 80 mg/d)with gradual tapering
Thomas (2020) [20]	26–52 weeks	Oral TAC was administered at a dose of 0.05 mg/kg/d (T0 levels of 6–8 ng/mL). In the event of inadequate clinical response at 8 weeks treatment, T0 levels was increased to 9–12 ng/ml. 12 weeks after achieving CR, the TAC doses were gradually reduced over 8 weeks and stopped	14–29 weeks	Oral steroid was administered at a dose of 1 mg/kg/d (a maximum dose of 60 mg/d). 1 week after achieving CR, the steroid dose was halved for 4–6 weeks then gradually reduced and stopped over a further 6 weeks

*T0* *levels* TAC trough levels

**Table 3:**
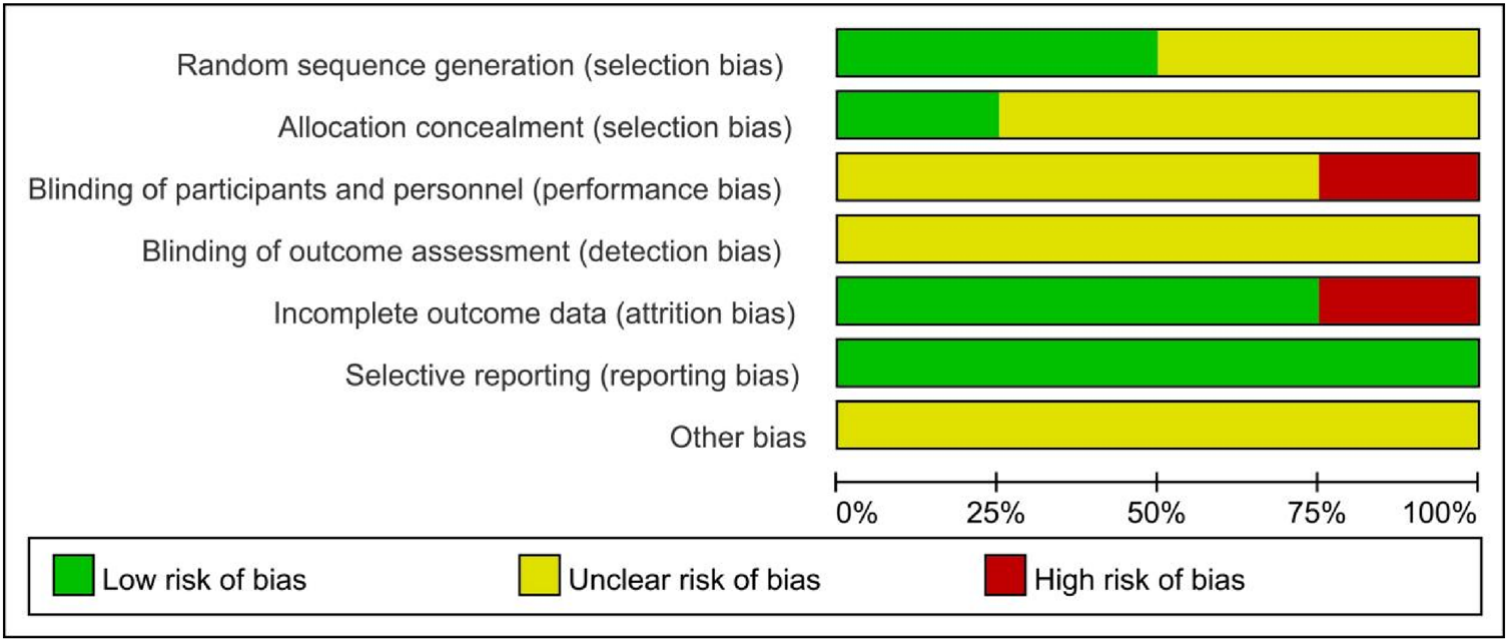
Quality assessment of RCTs

### Meta-analysis results

#### CR and TR

**Fig. 2:**
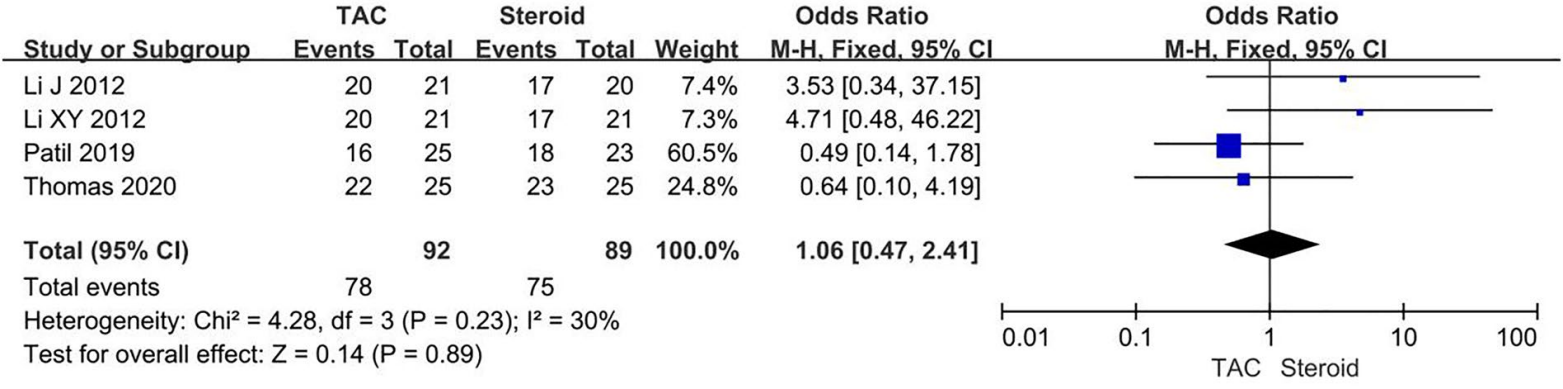
Forest plots comparing CR between TAC and steroid group

Data about CR were reported in eight articles, 78/92 (84.8%) for TAC group and 75/89 (84.2%) for steroid group. The heterogeneity among studies was not substantial (*P* = 0.23, *I*^2^ = 30%), so finally the fixed-effects model was used for the meta-analysis. There was no significant difference between two groups concerning CR (OR 1.06, 95% CI 0.47–2.41, *P* = 0.89) (Fig. 2).

**Fig. 3:**
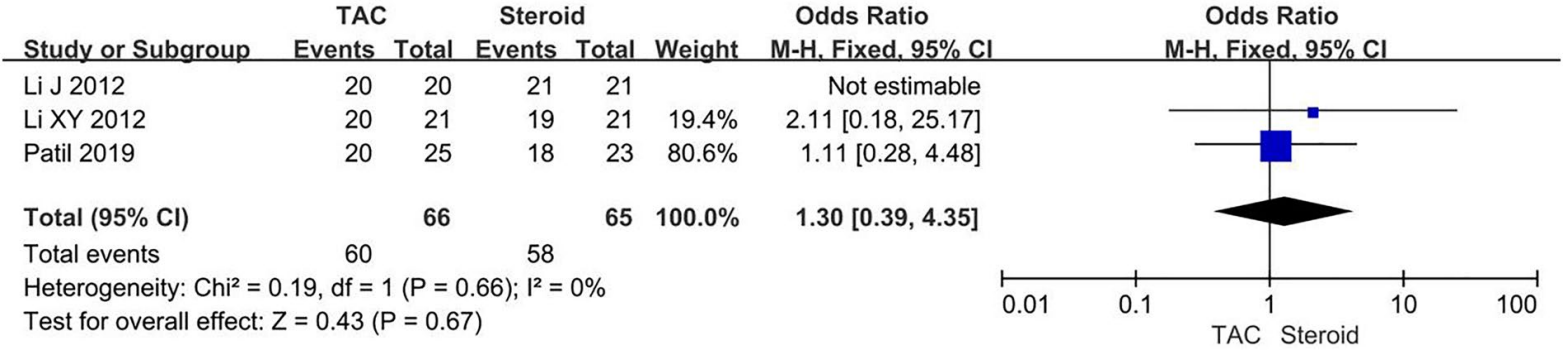
Forest plots comparing TR between TAC and steroid group

Data about TR were reported in three articles, 60/66 (90.9%) for TAC group and 58/65 (89.2%) for steroid group. The heterogeneity among studies was not substantial (*P* = 0.66, *I*^2^ = 0%), so finally the fixed-effects model was used for the meta-analysis. There was no significant difference between two groups concerning TR (OR 1.30, 95% CI 0.39–4.35, *P* = 0.67) (Fig. 3).

#### Relapse rate

**Fig. 4:**
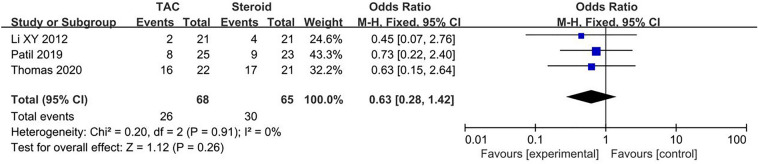
Forest plots comparing relapse rate between TAC and steroid group

Data about relapse rate were reported in three articles, 26/68 (38.2%) for TAC group and 30/65 (46.2%) for steroid group. The heterogeneity among studies was not substantial (*P* = 0.91, *I*^2^ = 0%), so finally the fixed-effects model was used for the meta-analysis. Relapse rate of steroid group was higher than TAC group, but the difference was not statistically significant (OR 0.63, 95% CI 0.28–1.42, *P* = 0.26) (Fig. 4).

#### Drug-related adverse effects

**Fig. 5:**
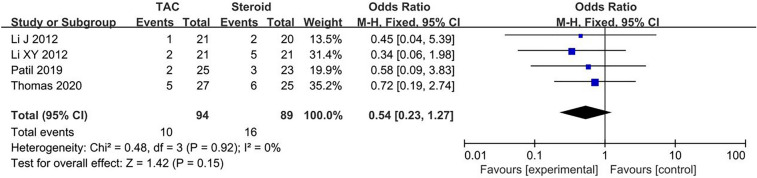
Forest plots comparing infection between TAC and steroid group

**Fig. 6:**
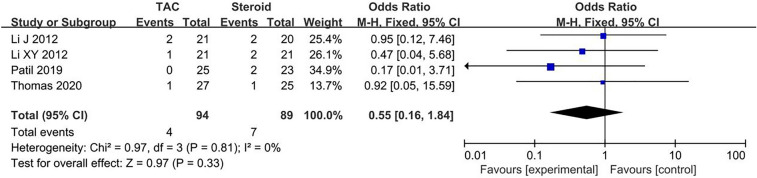
Forest plots comparing glucose intolerance between TAC and steroid group

**Fig. 7:**
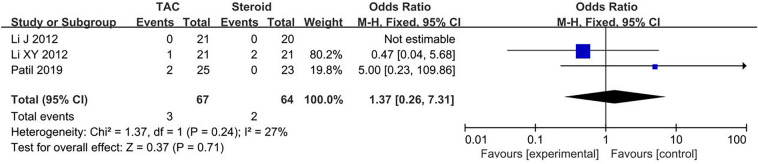
Forest plots comparing acute renal failure between TAC and steroid group

Data about main drug-related adverse effects were reported in four articles. Incidences of infection (10.6%, 10/94), glucose intolerance (4.3%, 4/94), acute renal failure (4.5%, 3/67), were in TAC group. Incidences of infection (18.0%, 16/89), glucose intolerance (7.9%, 7/89), acute renal failure (3.1%, 2/64), were in steroid group. There was no statistical significant difference between the two groups concerning infection (OR 0.54, 95% CI 0.23–1.27, *P* = 0.15), glucose intolerance (OR 0.55, 95% CI 0.16–1.84, *P* = 0.33) and acute renal failure (OR 1.37, 95% CI 0.36–7.31, *P* = 0.71). All forest plots of drug-related adverse effects are listed in Figs. 5, 6, 7.

### Sensitivity analyses

The sensitivity analyses for all outcomes after the two therapy regimens were used to judge the dependability of the results. We deleted one study at a time, the results of meta-analysis still showed no difference.

## Discussion

Corticosteroids are recommended as initial therapy of MCD, which is effective [6]. At the same time, the adverse effects of corticosteroids are significant, so the clinicians need consider alternative treatments. The current guidelines lack advice on the effectiveness of steroid-free regimens used as initial therapy of MCD. In some studies, TAC was reported to be effective in treatment of frequently relapsing, steroid-dependent, and steroid-resistant MCD [21–23]. Our meta-analysis was conducted to compare the efficacy and safety between TAC monotherapy with corticosteroid monotherapy for initial therapy of MCD. We found that TAC monotherapy is comparable with corticosteroid monotherapy for MCD concerning remission, relapse and drug-related adverse effects.

At present, corticosteroid monotherapy is effective in the treatment of MCD, which can achieve CR in 80% of patients with MCD [6]. Our meta-analysis showed that CR of corticosteroid monotherapy group was 84.2% and was comparable to the remission rates reported in other studies. In our meta-analysis TAC monotherapy also achieved high remission rates of 84.8%. In Thomas study included in our meta-analysis, treatment times of TAC or corticosteroid were relatively short, but CR of the two treatment method were both high and close to 90%. In general, TAC monotherapy was comparable to corticosteroid monotherapy concerning remission of NS.

MCD is easy to recur. Our meta-analysis showed that relapse rates were similar in corticosteroid and TAC treatment groups, which were lower than the 48–76% relapse rates reported in other studies [5, 9, 24, 25]. In Li Xiayu and Patil study included in our meta-analysis, the follow-up time was not long enough, which might not reflect the final relapse rates. Regimens of longer treatment time and higher dose may decrease relapse rates [20].

Long-term use of corticosteroid therapy can increase the incidence of drug-related adverse effects, so clinicians should evaluate the beneficial and adverse effects when prescribing treatment regimens for MCD patients. There are some significant unpleasant cosmetic drug-related adverse effects associated with corticosteroid such as obesity, acne, striae, and moon facies, which can be debilitating for patients, especially the young adults, and have an impact on adherence with treatment. Neither of these cosmetic drug-related adverse effects occurred in the TAC treatment. Concerning other common adverse effects, such as infection, glucose intolerance, acute renal failure, TAC and corticosteroid have no significant difference. The nephrotoxicity of TAC is of great concern. It has been reported in the literature that the nephrotoxicity of TAC is related to its dose and concentration. The initial dose of TAC was 0.15 mg/kg/d, which can lead to acute reversible nephrotoxicity. The initial dose of TAC was 0.08 mg/kg/d, which do not occur nephrotoxicity [26].

There were some limitations in our meta-analysis. First, there were some differences concerning the specific treatment regimen and definition of outcomes, which might affect the heterogeneity among the studies. Second, most included studies had small sample size and the number of included studies was small, so our meta-analysis may not be adequate to judge effectiveness and safety of the two treatment.

## Conclusions

Our meta-analysis revealed TAC monotherapy is comparable with corticosteroid monotherapy in initial therapy of MCD. To further confirm the conclusion, more large multicenter RCTs are necessary.
